# Investigating the molecular mechanism of iguratimod act on SLE using network pharmacology and molecular docking analysis

**DOI:** 10.3389/fbinf.2022.932114

**Published:** 2022-09-13

**Authors:** Huiqiong Zeng, Shuai Chen, Xiaoping Lu, Zhenbo Yan

**Affiliations:** ^1^ Shenzhen Futian Hospital for Rheumatic Diseases, Shenzhen, China; ^2^ Department of Traditional Chinese Medicine, Yunnan University of Traditional Chinese Medicine, Kunming, China

**Keywords:** iguratimod, systemic lupus erythematosus, network pharmacology, molecular docking, treatment

## Abstract

**Objective:** Iguratimod (*IGU*) is a novel small disease-modifying compound widely used in Asia for the treatment of rheumatic diseases. *IGU* is a methane sulfonanilide. We applied network pharmacology to investigate the pharmacological mechanisms of *IGU* act on SLE.

**Methods:** We used PharmMapper, UniProt, and OMIM databases to screen the potential targets of *IGU*, and the SLE-related disease targets were predicted. Hub target genes among the intersections of the potential targets (*IGU*) and related genes (SLE) were validated using the PPI network generated by the String database. GO and KEGG enrichment analyses were carried out using the David online platform. Finally, the molecular docking of hub targets and their corresponding compounds were completed through AutoDock Vina and PyMOL software for visualization.

**Result:** A total of 292 potential targets of *IGU*, 6501 related disease targets of SLE, and 114 cross targets were screened from the aforementioned database. Network topology analysis identified 10 hub targets, such as CASP3, AKT1, EGFR, MMP9, and IGF1. GO enrichment analysis mainly focuses on the negative regulation of the apoptotic process and signal transduction. KEGG enrichment analysis illustrated that the PI3K-AKT signaling pathway, MAPK signaling pathway, and FoxO signaling pathway might play a significant role in the pharmacological mechanisms of *IGU* act on SLE. Molecular docking confirmed that the *IGU* ligand had strong binding activity to the hub targets.

**Conclusion:** This study based on network pharmacology and molecular docking validation preliminarily revealed the protein targets affected by *IGU* acting on SLE through, and explored potential therapeutic mechanism role of *IGU* in SLE treatment by multi pathways.

## Introduction

Systemic lupus erythematosus (SLE) is a significant heterogeneous autoimmune disease characterized by the loss of immune tolerance and defective immune regulation (Kiriakidou and Ching, 2020). The etiology and pathogenesis of SLE are remained unclear. It is mainly caused by a complex interplay of genetics, environment, and hormones resulting in immune dysregulation, which leads to autoantibody production, inflammation, and destruction of end-organs (Pearce, 2016). A growing knowledge of pathogenesis of SLE enabled the research on novel therapeutic agents directed at specific disease-related targets (Bakshi et al., 2015).

Despite research studies in therapeutic approaches over the past few years, SLE still leads to relatively high morbidity and mortality increases. The long-term use of immunosuppressants for SLE might lead to serious adverse events, such as infections and toxicity. SLE still lacks safe and effective treatment (Fanouriakis et al., 2021).

In recent years, a new immunomodulatory drug, iguratimod, emerged as a potential candidate for the treatment of autoimmune diseases. It has been approved for the treatment of rheumatoid arthritis (RA) in Northeast Asia (Lü et al., 2008; Lu et al., 2009).

Iguratimod (*IGU*) is a novel immunomodulatory anti-rheumatic drug that is currently only approved in Japan and China (Mucke, 2012a).

The mechanisms of *IGU* in treating rheumatic diseases include inhibition of inflammation, immunomodulator, effective bone protection, and anti-pulmonary fibrosis (Du et al., 2008; Zhao et al., 2019).

There is evidence that *IGU* suppresses multiple inflammatory cytokines and chemokines such as interleukin (IL)-17, IL-6, and NF- κB activated (Hou et al., 2021; Liu et al., 2021; Xia et al., 2021), which are relevant to pathogenesis of SLE.

In recent years, many studies have pointed out that *IGU* may protect against SLE. An investigational treatment found that *IGU* showed positive results in an early clinical study in patients with refractory lupus nephritis (LN), meaning that it is recalcitrant to previous treatment with at least two immunosuppressive agents (Kang et al., 2020a). *In vivo*, *IGU* alleviated tubulo-interstitial lesions, especially tubulo-interstitial fibrosis in LN (Xue et al., 2022). *IGU* could attenuate the severity of nephritis in the murine lupus model (BALB/c mice) in a dose-dependent manner (Xia et al., 2021). However, the molecular mechanisms underlying *IGU* in the treatment of SLE remain poorly known.

**FIGURE 1:**
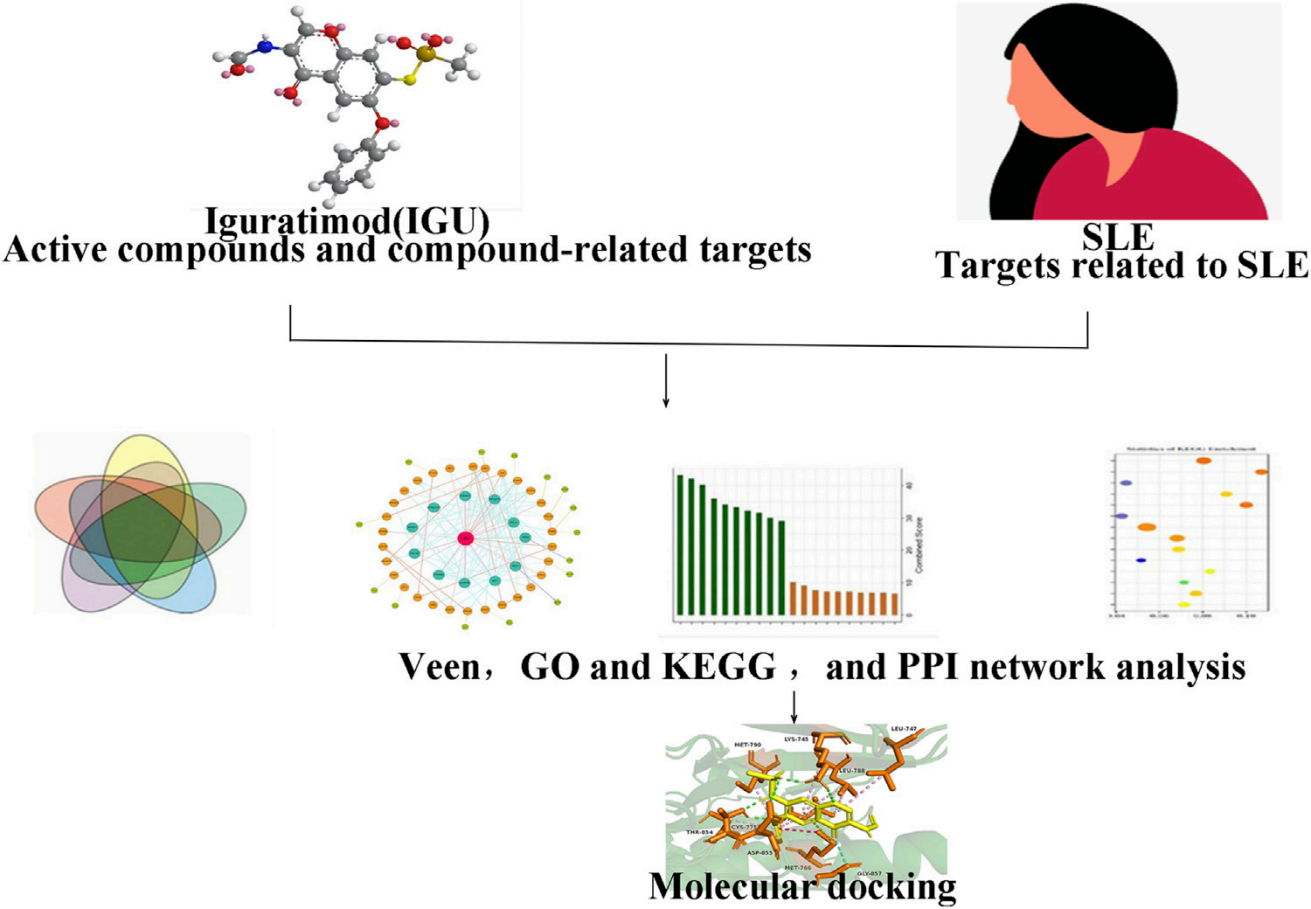
Study flow.

Network pharmacology is a research method to explore the complex association relationship between drugs and diseases based on the multidisciplinary techniques and knowledge such as systems biology, pharmacology, and molecular biology (Hopkins, 2007). In this study, will use network pharmacology and molecular structure strategies to initially elucidate the possible mechanism of *IGU* in the treatment of SLE by screening the main targets of *IGU* and the potential therapeutic targets in SLE and analyzing the active component target network (Figure 1).

## Methods

### Acquisition of components and targets of IGU

The PubChem database was used to obtain the molecular structure of *IGU* (SDF format), and potential action targets of *IGU* (CAS NO: 123663-49-0; molecular weight 374.37) were predicted by the PharmMapper database (http://www.lilab-ecust.cn/pharmmapper/). The PharmMapper server provided the molecular structures to obtain potential targets, and build a complete drug target database (Wang et al., 2017). The Swiss Target Prediction database (21) predicts targets from a combination of 2D and 3D structural similarities of known small molecule substances. The way PharmMapper identifies potential drug targets is mainly by mapping the reverse pharmacophore.

Then, the simultaneous selection of a Human Protein Target database is generated by optimizing compound structures for multiple structure analysis, in which parameter matching shub (fit shub>2) can be used as an adjudication criterion for the outcome of the *IGU* ligand (a small molecule) target screening.

The collected protein or gene information was normalized encoded in the UniProt database (https:// www.uniprot.org/).

### Acquisition of potential therapeutic targets of SLE and construction of Venn diagrams

We used “Systemic lupus erythematosus” as a keyword to collect SLE-related disease target genes in the OMIM (https://omim.org/ ), DisGeNet (https://www.disgenet.org/), and the GeneCards databases (https://www.genecards.org/, respectively. Then the intersection targets of *IGU* against SLE were mapped in the Venn 2.1.0 online platform (http://www.interactivenn.net/) to obtain the overlapping targets.

### Build protein–protein interaction network

The intersection targets of *IGU* and SLE were uploaded into the database of String (https://string-db.org), and the protein–protein interaction (PPI) network was constructed. The Cytoscape software (version 3.6.1) network (http://www.cytoscape.org;VERSION 3.6.1) was conducted to make visualization of the results. The top 10 disease–drug targets were finally obtained from the PPI network using Cytoscape plugin cytoHubba by the Maximal Clique Centrality (MCC) method (von Mering et al., 2003).

### Gene expression analysis

David (https://david.ncifcrf.gov/) was used to screen the differentially expressed gene (DEG) analysis. The fold change as well as the *p*-value was calculated with a t-test. The threshold set for DEGs was a fold change ≥ 2.0 and a *p*-value ≤ 0.05. GO and KEGG analyses were performed by selecting GO biological processes, GO molecular functions, GO cellular components, and KEGG pathway. GO and KEGG pathway enrichment results were plotted as bar graphs and bubble charts. Analyses of GO and KEGG were conducted.

### Network construction of SLE-IGU

We constructed a network of component-target-pathway diagram between *IGU* potential targets and SLE-related targets using the Cytoscape database for visualization analysis.

### Molecular docking prediction

#### Docking preparation and preprocessing

The molecular structure files of four target proteins, namely, AKT1 (PDB ID: 3o96), CASP3 (PDB ID: 1nmx), EGFR (PDB ID: 1ivo), and IGF1 (PDB ID: 1imx) with inhibitors were searched and downloaded by utilizing the PDB database (https://www1.rcsb.org/). The molecular structure file of *IGU* (PubChem CID: 124246) was downloaded using the PubChem database (https://pubchem.ncbi.nlm.nih.gov/). Downloaded target proteins were subjected to manipulation such as removing water molecules and removing protomers using Pymol software (2.3.0 version). QM calculations on the optimal conformation of the *IGU* ligand were executed in the ORCA software (5.03 version) package as implemented.

#### Molecular docking operation

AutoDock Vina (v.1.2.0 version) was used to verify the molecular docking between selected hub targets of *IGU* and that of SLE (Rizvi et al., 2013). PyMOL software was used to visualize the four sets of docking results and to contrast the interactions between the inhibitor molecule of the original protein complex and *IGU* ligand with the target proteins.

The docking scope is set within the active pocket where independent protomers of the target protein reside. Molecular docking binding energy was calculated by running AutoDockTools software (1.5.6 version) after grid and dock parameters were ready. The binding energy ≤0 kcal mol^−1^ implied that the compound could bind and interact with the hub target, while the binding energy <−5 kcal mol^−1^ indicated as a strong binding force. Figure 5 shows the docking results for the four groups.

## Result

### Target gene screening of IGU

**FIGURE 2:**
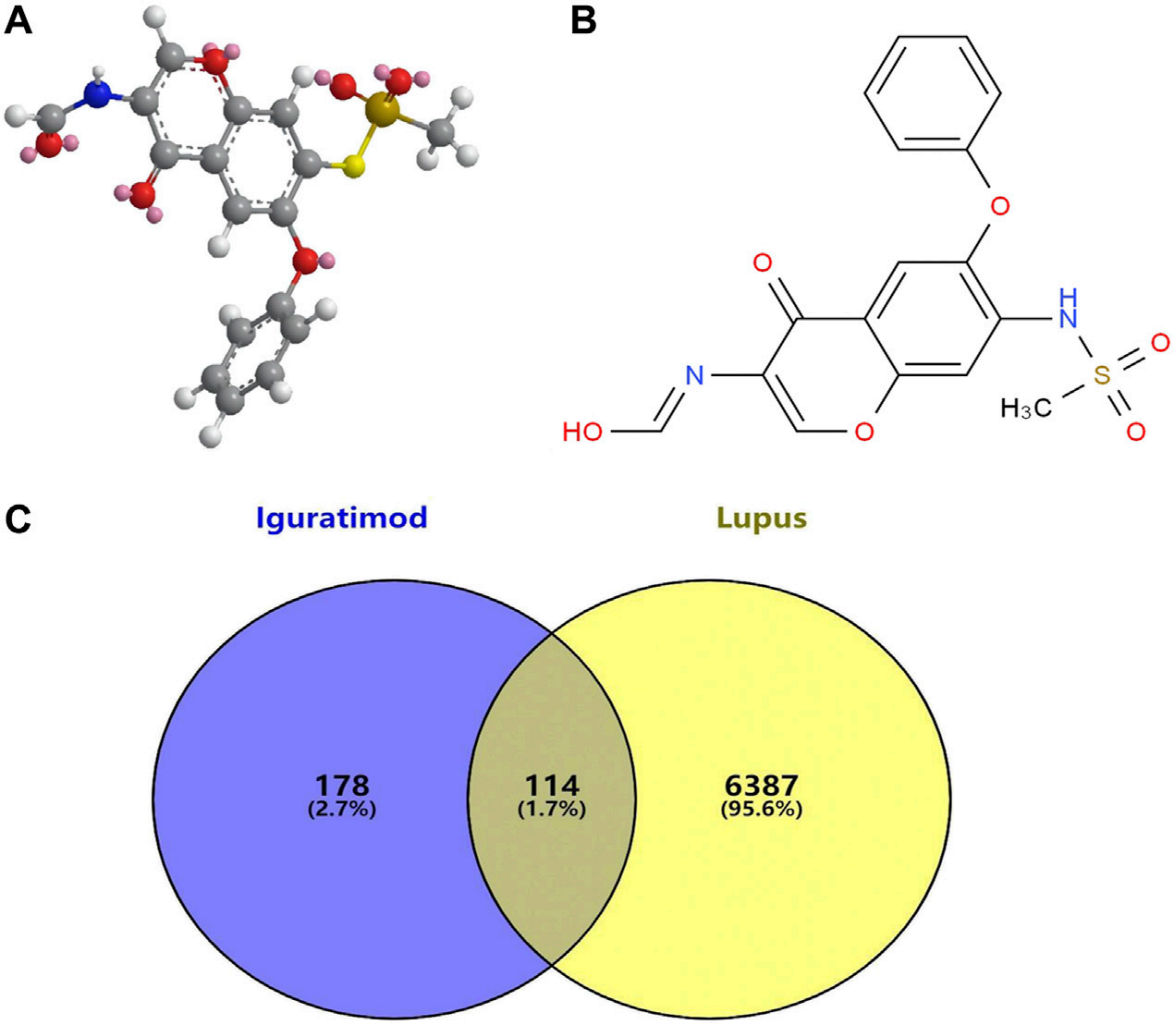
Associated targets predicted from databases involved in *IGU* (Ligand: 124246) and SLE. **(A)** 3D chemical structures of *IGU*. **(B)** 2D chemical structures of *IGU*. **(C)** Venn diagram of *IGU* targets and SLE.

Potential targets for *IGU* were predicted by the PharmMapper database. The 2D and 3D chemical structures of *IGU* are presented in Figure 2A. A total of 300 drug targets were finally obtained. After deleting duplicate targets, 292 targets and their abbreviations were obtained. Most of the potential targets of *IGU* are transferase (26%), hydrolase (25%), and oxidoreductaselyase (8%), as presented in Figure 2B.

### Potential targets of IGU in the treatment of SLE

A total of 54, 1883, and 6019 disease targets were obtained from three databases’ collection, and 6501 SLE-related targets were obtained after cleaning of duplicates. We imported SLE-related targets and *IGU*-related target protein genes into the online Venn platform to obtain the intersection genes, and a total of 114 intersection targets were obtained, as shown in Figure 2C.

### Construction of the PPI network

**FIGURE 3:**
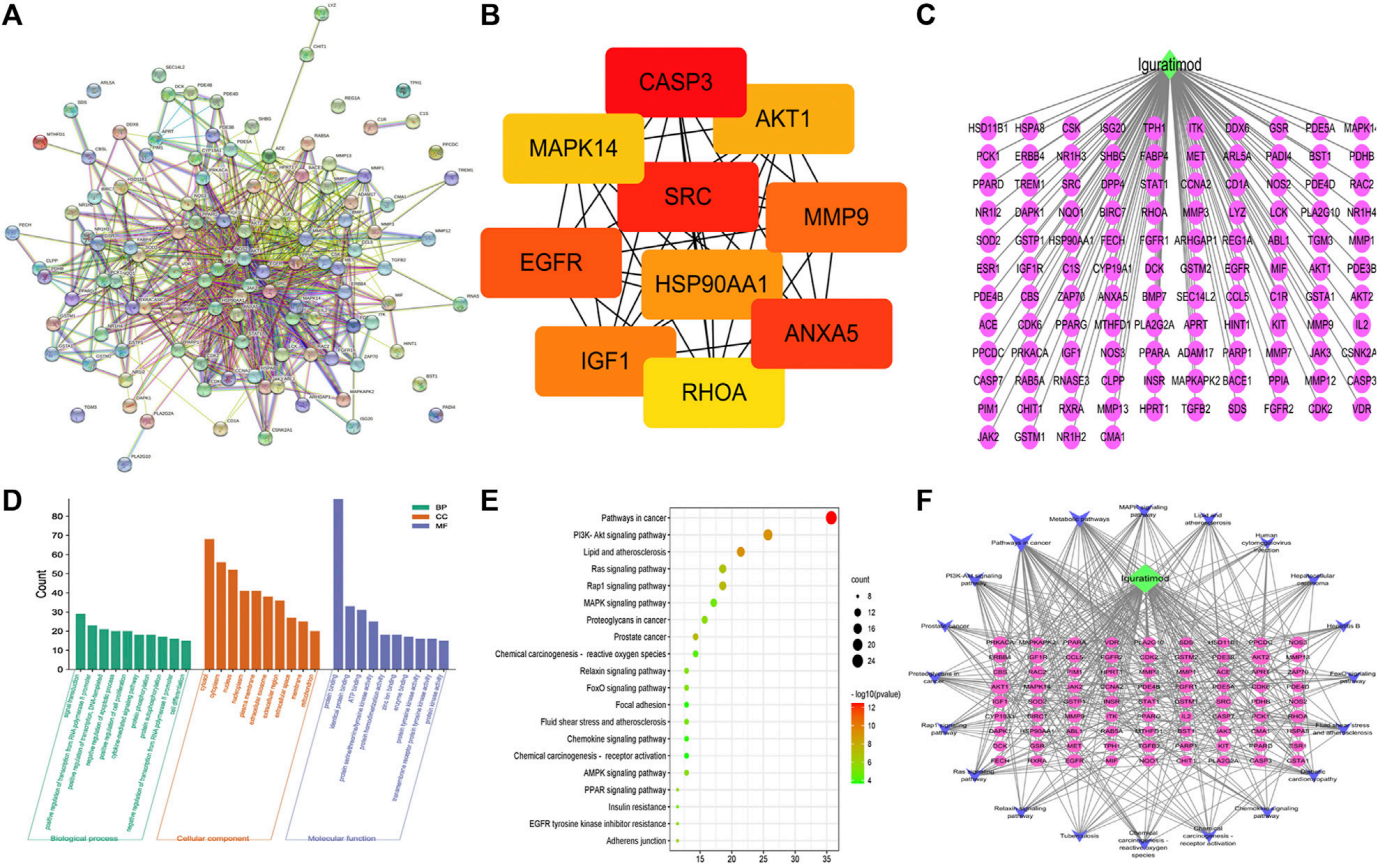
Bioinformatics analysis of overlapping targets. **(A)** Protein–protein interaction (PPI) network **(B)** Top 10 hub genes in the PPI network. (*C*) *IGU*-PACS potential target network. **(D)** Bar chart of GO enrichment analysis of the hub target of *IGU* in the treatment of SLE (the top 10 items); (green) biological processes, (orange) cell components, and (purple) molecular functions. **(E)** Bubble chart of KEGG analysis of the hub target of *IGU* in the treatment of SLE (the top 20 items). **(F)** Network of KEGG (the top 20 items) signaling pathway genes; (blue) pathway, (green) *IGU*, (yellow) four hub genes relevant to pathways and SLE, and (purple) potential target.

Using the String database, a protein–protein interaction (PPI) network of the 114 intersection targets was then performed to predict the functionality of interacting proteins and genes. Cluster analysis of the PPI network of genes significantly altered the expression (FC > 1.2 and *p* < 0.05). The PPI network consisted of 114 nodes and 763 edges (Figure 3A). Furthermore, the cytoHubba plugin was used to select the top 10 hub gene targets from the PPI network using the MCC method. The top 10 hub gene targets are CASP3, AKT1, SRC, MMP9, EGFR, ANXA5, IGF1, MAPK14, RHOA, and HSP90AA1 according to a degree in cytoHubba, and details are shown in Figure 3B;

### GO and KEGG pathway analyses of the related proteins

The GO and KEGG pathway analyses are performed to illuminate the top 10 molecular functions (MFs), biological processes (BPs), and cellular components (CCs). The result of GO showed that the potential targets of *IGU* have influence on the negative regulation of the apoptotic process, chaperone-mediated autophagy, identical protein binding, and ATP binding (Figure 3D) while the KEGG pathway enrichment analysis found that these targets were closely related to the PI3K-AKT signaling pathway, MAPK signaling pathway, FoxO signaling pathway, and chemokine signaling pathway, etc., (Figure 3E).

### Network construction of SLE-IGU with PACs and KEGG pathway genes

**FIGURE 4:**
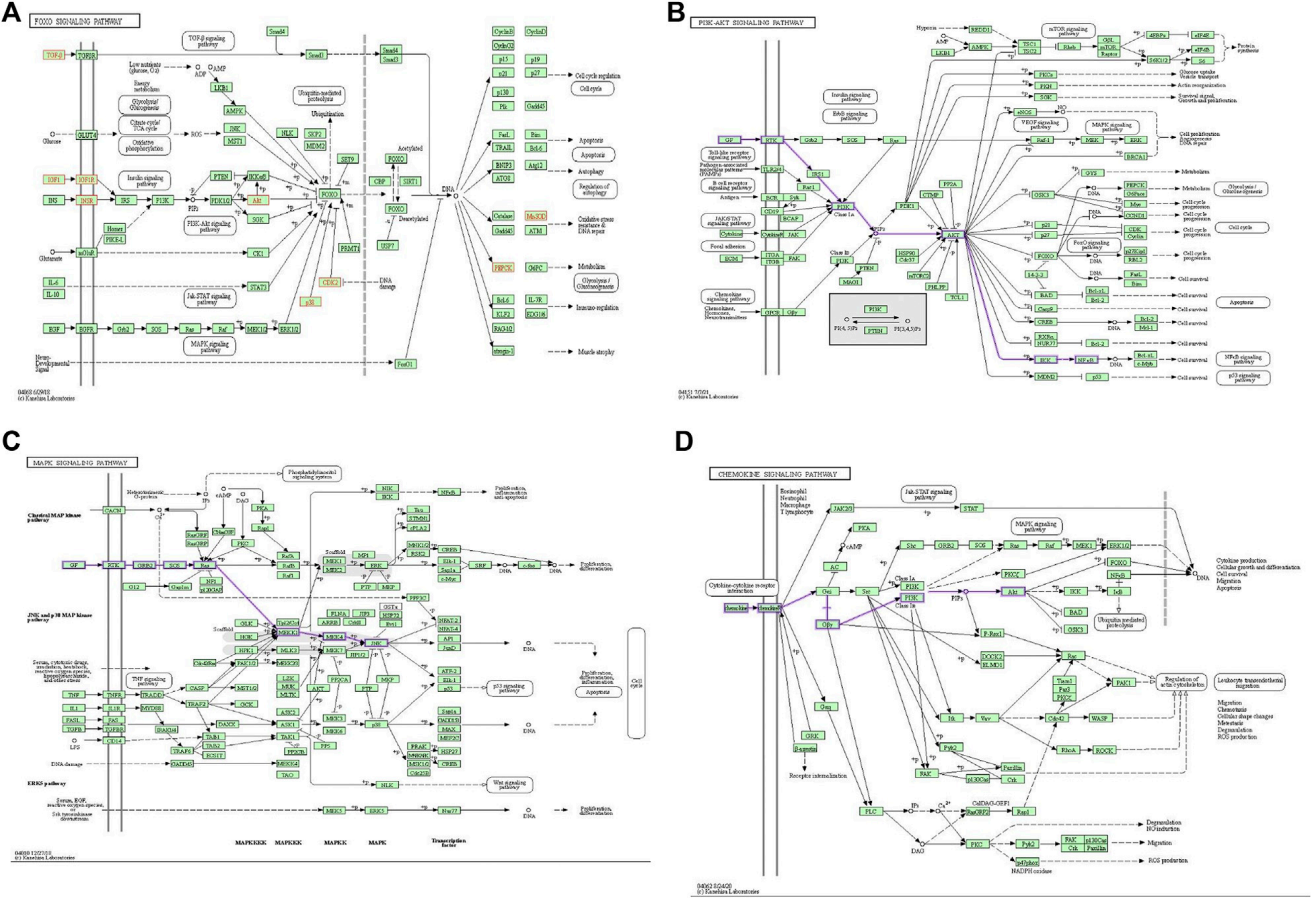
Pictures of KEGG gene pathway is displayed. **(A)** FoxO signaling pathway; (yellow node) genes existing in this *IGU* and SLE network. **(B)** PI3K/AKT signaling pathway; (purple edge) road map of genes associated with our network. **(C)** The MAPK signaling pathway; (purple edge) road map of genes associated with our network. **(D)** Chemokine signaling pathway; (purple edge) road map of genes associated with our network.

We mapped the *IGU*-PAC potential target using Cystoscape software of the network diagram, and the ingredient potential target network contains 115 nodes (including 114 target genes and 1 drug) and 114 edges (Figure 3C). Furthermore, the network of KEGG signaling pathway genes was constructed using Cytoscape software, which was associated with the top 20 pathways with their related target genes by KEGG analysis. The network diagram was found to have 102 nodes, including 81 common target genes, 1 drug, 1 disease, and 400 edges, as shown in Figure 3F. As illustrated in the figure, AKT, IGF1, and EGFR targets are involved in the FoxO signaling pathway and PI3K-AKT signaling pathway; AKT, IGF1, CASP3, and EGFR targets are tied to the MAPK signaling pathway, additionally. Based on KEGG results and association between *IGU* act on SLE, we constructed a target interaction graph to demonstrate the regulation of four target pathways with *IGU* in SLE (Figure 4).

### Molecular docking

Molecular docking analysis is a way of combining structural molecular biology and computer-aided drug design to evaluate the affinity between a small molecule ligand of a drug and a large molecule of a receptor or drug design (Meng et al., 2011).

We searched the PDB database for the four target protein structures with inhibitors and set the docking site of the *IGU* ligand to the protein according to where the inhibitor is located, so that *IGU* is docked in the active pocket of the protein where the inhibitor is located. By contrast, the *IGU* ligand and inhibitor molecule of the original protein complex were found to bind to the same amino acid residues in the docking results of four target proteins AKT1, CASP3, EGFR, and IGF1.

**FIGURE 5:**
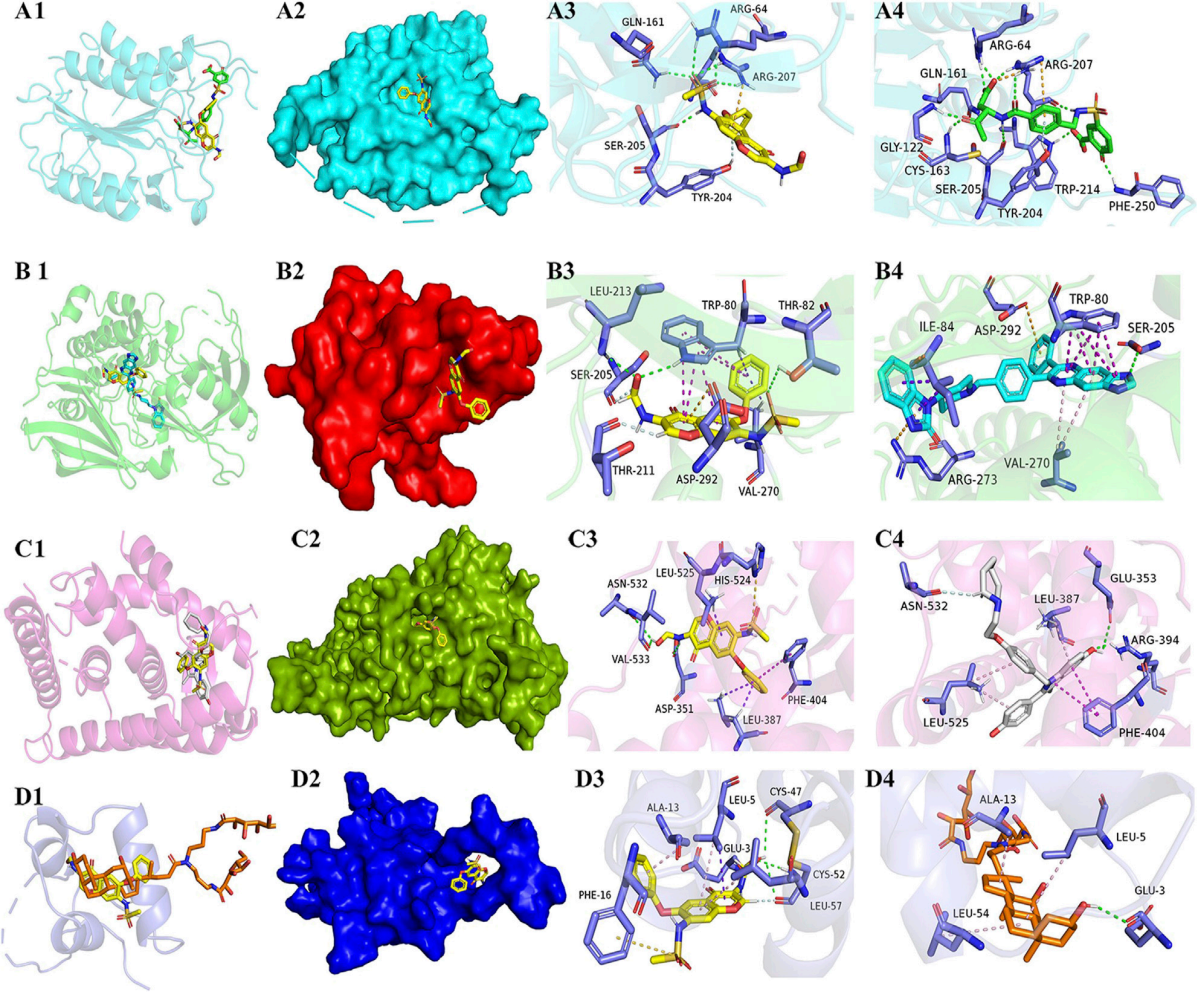
Diagrams of docking models of *IGU* and the four most related target molecules. 5–1. **(A–D)** Docking views of target proteins CASP3, AKT1, EGFR, and IGF1 with *IGU*, respectively. 5–2. 1, 2, 3, and 4 in each line indicates the description of the following, respectively: 1) Number 1 of each group is a cartoon comparison plot of the binding of *IGU*, inhibitor, and the target protein, with *IGU* as a yellow structure, and the four groups of inhibitors colored green, light blue, white, and orange, respectively. 2) Number 2 of each group is a surface modeling of number 2, respectively. 3) Number 3 of each group is a concrete picture of the binding of *IGU* to the target protein, respectively. 4) Number 4 of each group is a concrete picture of inhibitor binding to the target protein, respectively. **(A)** *CASP3*-*IGU*. **(B)** *AKT1*-*IGU*. **(C)** *EGFR*-*IGU*. **(D)** *IGF1*-*IGU*.

The results of binding energy, the number of hydrogen bonds formed between the docking of the target protein and *IGU* ligand, and the amino acid residues of the *IGU* ligand that had a common effect with the original inhibitor and the target protein are shown in Table 1, and 3D structures are shown in Figure 5. The binding energy <−5 kcal·mol-1 indicated as a strong binding force.

**TABLE 1:** Molecular docking score.

Hub target	PDB ID	Spacing	Docking score (kcal/mol)
EGFR	6tG0	0.500	−8.89
CASP3	1 nms	0.375	−8.62
AKT1	3cqu	0.375	−7.73
IGF1	1pmx	0.375	−6.85

As shown in Figure 5B 3/4, the *IGU* small molecule forms three conventional hydrogen bonds with amino acid residues trp-80 (2.48 Å), thr-82 (3.28 Å), and leu-213 (3.04 Å) in the AKT1 target protein. It forms two carbon–hydrogen bond interactions (carbon–hydrogen bond) with ser-205 (2.46 Å) and thr-211 (2.62 Å). It forms five π–π interactions with trp-80 (4.33 Å, 4.50 Å, 5.26 Å, 5.71 Å, and 5.79 Å). Likewise, it also forms two π with trp-80 (2.55 Å) and val-270 (2.55 Å)-σ effect. The *IGU* small molecule forms one π–ion bond interaction with asp-292 (4.24 Å). The inhibitor small molecule forms one conventional hydrogen bond interaction with amino acid residue ser-205 (2.89 Å) in the AKT1 target protein. It forms six π–π interactions with trp-80 (3.68 Å, 3.73 Å, 4.06 Å, 4.17 Å, and 5.36 Å), and two π–ion bond interactions with arg-273 (4.20 Å) and asp-292 (4.75 Å), respectively. It forms two π with ile-84 (4.42 Å and 4.42 Å)-σ effect and two π–alkyl interactions with val-270 (5.20 Å and 5.26 Å), respectively. The alignments found that both *IGU* and the inhibitor bound to the same amino acid residues in the docking results for AKT1 target proteins, such as thr-80, ser-205, val-270, and asp-292.n bond. The binding sites of *IGU* to the four target proteins were nearly the same as that of the inhibitor, and the binding energy of *IGU* to the four target proteins was all excellent, and the binding force was abundant. Thus, *IGU* used in this experiment is likely to exert inhibitory effects through docking with four target proteins.

## Discussion

Systemic lupus erythematosus (SLE) is an autoimmune disease, with systemic clinical manifestations including the skin rash, brain, kidneys, heart, and lungs (Hoi and Morand, 2021). Abnormal immune response, inflammation, autophagy, and apoptosis are the key pathogenesis of SLE (Tsokos et al., 2016; Feng et al., 2018). Novel therapy agents can provide more options in the armamentarium for treating this complex disease, but ongoing studies are needed to improve understanding of the optimal treatment algorithm to maintain the treatment effect for SLE patients.

Iguratimod (*IGU*) has been found to be effective in the treatment of SLE (Yan et al., 2014; Kang et al., 2020b). Clinical and experimental evidence showed that *IGU* can reduce immunoglobulin production by acting directly on B lymphocytes in mice and humans (Tanaka et al., 2003). The immunomodulatory effect of *IGU* is unknown, but may depend in part on its effect on nuclear factor-κB (NF-κB) or other cell signaling pathways (Mucke, 2012b). *IGU* can also inhibit apoptosis of PBMC (Liu et al., 2014), which may treat SLE through these effects, but the hub targets and significant pathways of *IGU* against SLE are still unclear.

Network pharmacology (NP), as an emerging drug research strategy, has gained wide applications in drug research, enabling the description of relationships among biological systems, drugs, and diseases from a network perspective, such as SLE (Zhang et al., 2019; Xinqiang et al., 2020). The aim of this study was to perform a preliminary evaluation of *IGU* as a possible mechanism of drugs in SLE patients based on NP analysis.

In this study, we constructed an *IGU*-targeted SLE-related gene network consisting of 114 targets. GO and KEGG analyses showed that *IGU* could regulate immune, apoptosis, and autophagy signaling pathways. We further obtained 10 hub targets from 114 cross targets by constructing the PPI network and compound–target–disease network analysis. We selected four targets closely related to SLE together with a higher node degree in the network (CASP3, AKT1, EGFR, and IGF1), and performed molecular docking to verify the interaction between *IGU* and the four targets. Our findings demonstrated the effectiveness of *IGU* in SLE treatment from a bioinformatics perspective and provide possible pharmacological multi-targets and multi-pathway mechanisms of *IGU*. The results may also facilitate targeted drug design and basic research for SLE in the future.

According to our finding of NP, AKT1, CASP3, EGFR, and IGF1 played critical roles in the treatment of SLE with *IGU*. These target proteins are involved in oxidative stress, inflammation, angiogenesis, and immunomodulator. Based on the previous reports, AKT1 serine/threoe kinase is a key autophagy target of the PI3K signaling pathway, and plays a role in the differentiation of peripheral B cells and in T-cell homeostasis in SLE patients (Juntilla and Koretzky, 2008; Garcia-Rodriguez et al., 2012). In another study, it was confirmed that PI3kp110, AKT, and mTOR activated the expression of Fox 3 in CD4^+^ thrombocytes and peripheral T cells through T-cell receptor control (Sauer et al., 2008). CASP3 contributes to apoptotic pathways. It was found that CASP3 inhibitors could normalize T-cell function in SLE (Krishnan et al., 2005). The expression of activated CASP3 was correlated with various markers of activity of lupus nephritis (LN) and was known as a marker of apoptosis in LN (Jeruc et al., 2006).

Epidermal growth factor receptor (EGFR) itself has tyrosine kinase activity and, once combined with EGF, can initiate related genes in the nucleus, thereby promoting cell division and proliferation (Costa-Reis et al., 2015). A single center study found that polymorphisms in the *EGFR* gene are markers of susceptibility or disease activity in Taiwanese patients with SLE (Huang et al., 2004). Activated EGFR upregulates ROS production and endoplasmic reticulum stress, and this mechanism plays an important role in the pathogenesis of lupus nephritis (Liu et al., 2020).

Insulin-like growth factor 1 (IGF-1) is a hormone, which stimulates the growth, differentiation, and metabolism of various cell types, and induces angiogenesis (Joseph D'Ercole and Ye, 2008). IGF-1 stimulates the expression of angiogenesis-related growth factors by activating the PI3K/Akt signaling pathway (Lin et al., 2017). In elderly patients with SLE, IGF1 levels might provide a clinical marker of disease activity (Stylianou et al., 2011). Serum levels of angiogenic activity were elevated in patients with SLE, and anti-angiogenic therapy may be a potentially effective and promising treatment for SLE (Liu et al., 2015).

Following GO and KEGG enrichment analyses, it was hypothesized that *IGU* has an impact on several key pathways that are important in the treatment of SLE. AKT1, CASP3, EGFR, and IGF1 may be targets for the action of *IGU* in the treatment of SLE.

An increasing number of studies have reported that the PI3K/Akt signaling pathway is involved in the pathogenesis of SLE. The expression of the PI3K/AKT signaling pathway is upregulated in murine lupus nephritis (Stylianou et al., 2011). The AMP-activated protein kinase (AMPK) activation inhibitor can attenuate inflammation in mesangial cells of the MRL/lpr mice model by inhibiting the PI3K/AKT pathway (Peairs et al., 2009). PI3K binding to EGFR can activate AKT, which in turn inhibits the activity of a series of downstream substrates, such as apoptosis-related protein CASP9, and the IGF-1/PI3K/AKT signal transduction pathway is involved in aging of many organisms (Longo and Finch, 2003). The enrichment analysis of this study showed that the PI3K/Akt signaling pathway may be significantly affected by *IGU* in SLE treatment. P38 MAP kinase (MAPK) participates in a signaling cascade controlling cellular responses to cytokines and stress. The MAPK signal pathway is known to contribute to the inhibition of nuclear factor-κB (NF-κB), which may be a potential intracellular mechanism leading to excessive lymphocyte activity in SLE (Wong et al., 2009). FoxO plays a critical role in regulating immune cell homeostasis in some disease states such as inflammatory arthritis and SLE through fundamental roles in T cells and B cells. The FoxO signaling pathway is involved in many cellular physiological events such as apoptosis, cell-cycle control, oxidative stress resistance, and longevity (Peng, 2007). The PI3K/AKT/FoxO3a pathway seems to play an important role in the kidney of MRL/lpr mice. Upregulation of FoxO3a expression achieved by inhibition of the PI3K/Akt pathway concomitantly improved lupus nephritis activity in MRL/lpr mice (Zhao et al., 2020).

Some studies had reported that chemokines and their receptors (CXCR) were expressed in several types of cutaneous damages associated with SLE (Lacotte et al., 2009). The phagocytosis of apoptotic cells in the chemokine signaling pathway may lead to a pro-inflammatory response in the presence of autoantibodies. This may sustain inflammatory conditions and the pathology found in overt lupus (Bolouri et al., 2022). This is the first study that has predicted the therapeutic mechanisms of *IGU* in SLE by using network pharmacology and molecular docking. The aforementioned findings were compatible with the results of GO enrichment analysis and KEGG analysis in this study.

To further verify the mechanism of action, we used molecular docking to analyze the binding energy of *IGU* ligand and the corresponding active targets. We also conducted the docking control of molecular docking between the target protein and the inhibitor of the reference compound. We performed the docking control of the target protein with the inhibitor of the reference compound for further validation. Our study provides a reference for the molecular docking results, space and energy matching correlation between molecules, the chemical compound of *IGU*.

Meanwhile, molecular docking showed that *IGU* had strong binding activity against four hub targets with all binding affinities below—5 kcal/mol, and the binding conformation was steady. For example, CASP3 is a representative apoptotic cytokine and has a good binding affinity with the *IGU* ligand, which also confirms that *IGU* may play an anti-apoptotic role in SLE.

In summary, the inhibitory effect of *IGU* with hub targets act on SLE may be involved in the immune cell regulation process, apoptosis, regulating immune cell function, angiogenesis, and endoplasmic reticulum stress through the regulation of MARK, PI3K/AKT, FoxO, and chemokine signaling pathways.

These studies are consistent with the findings of the present study, and to some extent demonstrate the reliability of the bioinformatic research approach, and the study of *IGU* targeting these signaling pathways may help to attenuate disease processes such as inflammation and immune disorders in SLE.

There are a few limitations in this study which are as follows: it is essential to evaluate the effect of *IGU* binding to target proteins, and molecular dynamics (MD) simulations, mass spectrometry (MS) analysis, and high-throughput nanoparticle assays (HMSA) are needed. Given the objective reasons such as the funding support of this study and the limitation of laboratory conditions, this aspect could be a fun and good warm-up for the latter part of further works *in vitro* to validate our findings, including MS and high-performance liquid chromatography (HPLC). Also, it is necessary to carry out further studies with the multi center.

## Conclusion

To sum up, we speculate that the mechanism of action of *IGU* on SLE may be exerted through the hub targets described earlier and the related protein, especially in NF-κB, MAPK, PI3K/AKT, inhibition of abnormal apoptosis, and other pathways. These findings expand our understanding of the mechanism of *IGU* in the treatment of SLE. *IGU* might act as a multicomponent, multi-target, and multi-pathway therapeutic agent for SLE.

## Data Availability

The datasets presented in this study can be found in online repositories. The names of the repository/repositories and accession number(s) can be found in the article/Supplementary Material.
